# CD40L modulates transcriptional signatures of neutrophils in the bone marrow associated with development and trafficking

**DOI:** 10.1172/jci.insight.148652

**Published:** 2021-08-23

**Authors:** Tábata Takahashi França, Ashraf Al-Sbiei, Ghada Bashir, Yassir Awad Mohamed, Ranieri Coelho Salgado, Lucila Akune Barreiros, Sarah Maria da Silva Napoleão, Cristina Worm Weber, Janaíra Fernandes Severo Ferreira, Carolina Sanchez Aranda, Carolina Prando, Mayra B. de Barros Dorna, Igor Jurisica, Maria J. Fernandez-Cabezudo, Hans D. Ochs, Antonio Condino-Neto, Basel K. Al-Ramadi, Otavio Cabral-Marques

**Affiliations:** 1 Department of Immunology, Institute of Biomedical Science, University of São Paulo, São Paulo, São Paulo, Brazil.; 2 Department of Medical Microbiology and Immunology, College of Medicine and Health Sciences, United Arab Emirates (UAE) University, Al Ain, Abu Dhabi, United Arab Emirates.; 3 Pediatric Allergy & Immunology Clinic, Caxias do Sul, Rio Grande do Sul, Brazil.; 4 Albert Sabin Hospital, Fortaleza, Ceará, Brazil.; 5 Division of Allergy, Immunology, and Rheumatology, Department of Pediatrics, Federal University of São Paulo, São Paulo, São Paulo, Brazil.; 6 Faculdades Pequeno Príncipe, Pelé Pequeno Principe Research Intitute, Curitiba, Paraná, Brazil.; 7 Hospital Pequeno Príncipe, Curitiba, Paraná, Brazil.; 8 Division of Allergy and Immunology, Department of Pediatrics, Children’s Institute, Hospital das Clínicas, São Paulo, São Paulo, Brazil.; 9 Osteoarthritis Research Program, Division of Orthopedic Surgery, Schroeder Arthritis Institute, University Health Network, Krembil Research Institute, University Health Network, Departments of Medical Biophysics and Computer Science, University of Toronto, Toronto, Ontaro, Canada.; 10 Institute of Neuroimmunology, Slovak Academy of Sciences, Bratislava, Slovakia.; 11 Department of Biochemistry and Molecular Biology, College of Medicine and Health Sciences, UAE University, Al Ain, Abu Dhabi, United Arab Emirates.; 12 Department of Pediatrics, University of Washington School of Medicine, and Seattle Children’s Research Institute, Seattle, Washington, USA.; 13 Zayed Center for Health Sciences, UAE University, Al Ain, Abu Dhabi, United Arab Emirates.; 14 Department of Clinical and Toxicological Analyses, School of Pharmaceutical Sciences, University of São Paulo, São Paulo, São Paulo, Brazil.; 15 Network of Immunity in Infection, Malignancy, and Autoimmunity (NIIMA), Universal Scientific Education and Research Network (USERN), São Paulo, São Paulo, Brazil.

**Keywords:** Cell Biology, Immunology, Bone marrow differentiation, Neutrophils

## Abstract

Neutrophils are produced in the BM in a process called granulopoiesis, in which progenitor cells sequentially develop into mature neutrophils. During the developmental process, which is finely regulated by distinct transcription factors, neutrophils acquire the ability to exit the BM, properly distribute throughout the body, and migrate to infection sites. Previous studies have demonstrated that CD40 ligand (CD40L) influences hematopoiesis and granulopoiesis. Here, we investigate the effect of CD40L on neutrophil development and trafficking by performing functional and transcriptome analyses. We found that CD40L signaling plays an essential role in the early stages of neutrophil generation and development in the BM. Moreover, CD40L modulates transcriptional signatures, indicating that this molecule enables neutrophils to traffic throughout the body and to migrate in response to inflammatory signals. Thus, our study provides insights into the complex relationships between CD40L signaling and granulopoiesis, and it suggests a potentially novel and nonredundant role of CD40L signaling in neutrophil development and function.

## Graphical abstract

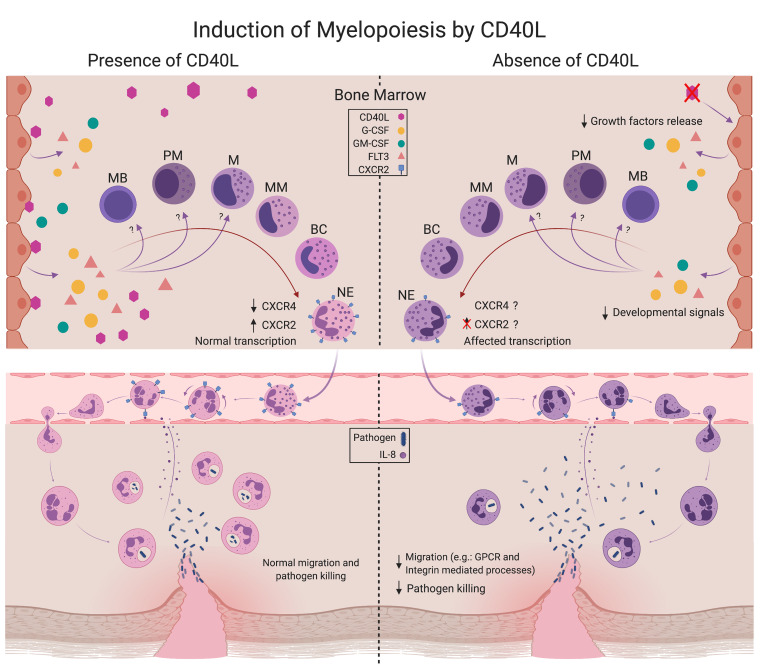

## Introduction

Neutrophils are myeloid cells representing the most abundant subset of leukocytes in the human blood (1). They are powerful innate immune effector cells, destroying pathogens by phagocytosis, degranulation, reactive oxygen species (ROS) production, and neutrophil extracellular trap (NET) release (2, 3). In steady-state conditions, approximately 0.5 × 10^11^ to 1 × 10^11^ neutrophils are generated daily in an adult (1). The production of neutrophils is the major activity of the BM since almost two-thirds of the hematopoiesis is dedicated to myelopoiesis, namely the production of granulocytes (neutrophils, eosinophils, and basophils) and monocytes (4). The homeostasis of neutrophil production and distribution throughout the body is maintained by the balance between granulopoiesis, BM storage and release, intravascular transit and margination of neutrophils through specific organs, and finally clearance and removal of aged neutrophils in the spleen, BM, and other organs (4–6).

Neutrophils develop in the BM from hematopoietic stem cells — a process called granulopoiesis. Myeloid progenitors differentiate sequentially into myeloblasts, promyelocytes, myelocytes, metamyelocytes, band cells, and finally mature neutrophils (4), which are released into the bloodstream with the capacity to perform their effector functions for maintenance of homeostasis and control of infections (2, 7). Several myelopoiesis-promoting growth factors, such as granulocyte-colony stimulating factor (G-CSF), granulocyte-macrophage CSF (GM-CSF), and FMS-like tyrosine kinase 3 ligand (FLT3L) act in the early stages of hematopoiesis, inducing and controlling the commitment and maturation of progenitor cells to neutrophils (4, 8–12).

CD40 ligand (CD40L) is mainly known by its role in antibody production (i.e., induction of class-switch recombination and immunoglobulin somatic hypermutation) (13–15) when expressed by activated CD4^+^ T cells. However, in vitro studies have demonstrated that soluble CD40L (sCD40L), a molecule produced by T cells (16) and platelets (17), also participates in hematopoiesis (18–20). In line with these findings, it has been shown that the interaction between CD40L and its receptor CD40 influences the generation of neutrophils under steady-state conditions (21). This effect of the CD40L-CD40 interaction on myeloid cell development occurs directly by activating myeloid cell progenitors and indirectly by modulating local stromal production of myeloid growth factors such as G-CSF, GM-CSF, thrombopoietin, and FLT3L (19, 22–24), indicating that CD40L modulation of myeloid cell development is a complex process that involves a network of several cells and signaling pathways.

Of note, CD40L-deficient patients suffer from life-threatening infections caused by several classes of microorganisms (25–27), which are attributed not only to B and T cell defects (13, 28–30), but also to functional defects of DCs (31), monocyte/macrophages (32), and neutrophils (33). Interestingly, neutropenia is the most common noninfectious manifestation in CD40L deficiency, affecting approximately 70% of patients (34, 35). Neutropenia can present as episodic, cyclic, or chronic presentation, contributing to greater vulnerability to serious and recurrent infections in CD40L-deficient patients (36).

Despite these clinical observations suggesting a critical role of CD40L-CD40 interaction in the development of neutrophils, the molecular mechanisms by which CD40L-CD40 interaction regulates neutrophil development remains undetermined. Neutrophils have been implicated in different biological processes (BPs), and dysregulation of neutrophil transcription profiles plays an essential role in the pathologies of cancer (37), congenital neutropenia (38), infections (39), and autoimmune diseases (40, 41). Here, we present data indicating that CD40L modulates transcriptional signatures of neutrophils in the BM associated with development and trafficking, suggesting a potentially novel and nonredundant role of CD40L signaling in neutrophil development and function.

## Results

### CD40L promotes myelopoiesis and granulopoiesis in vivo.

**Figure 1:**
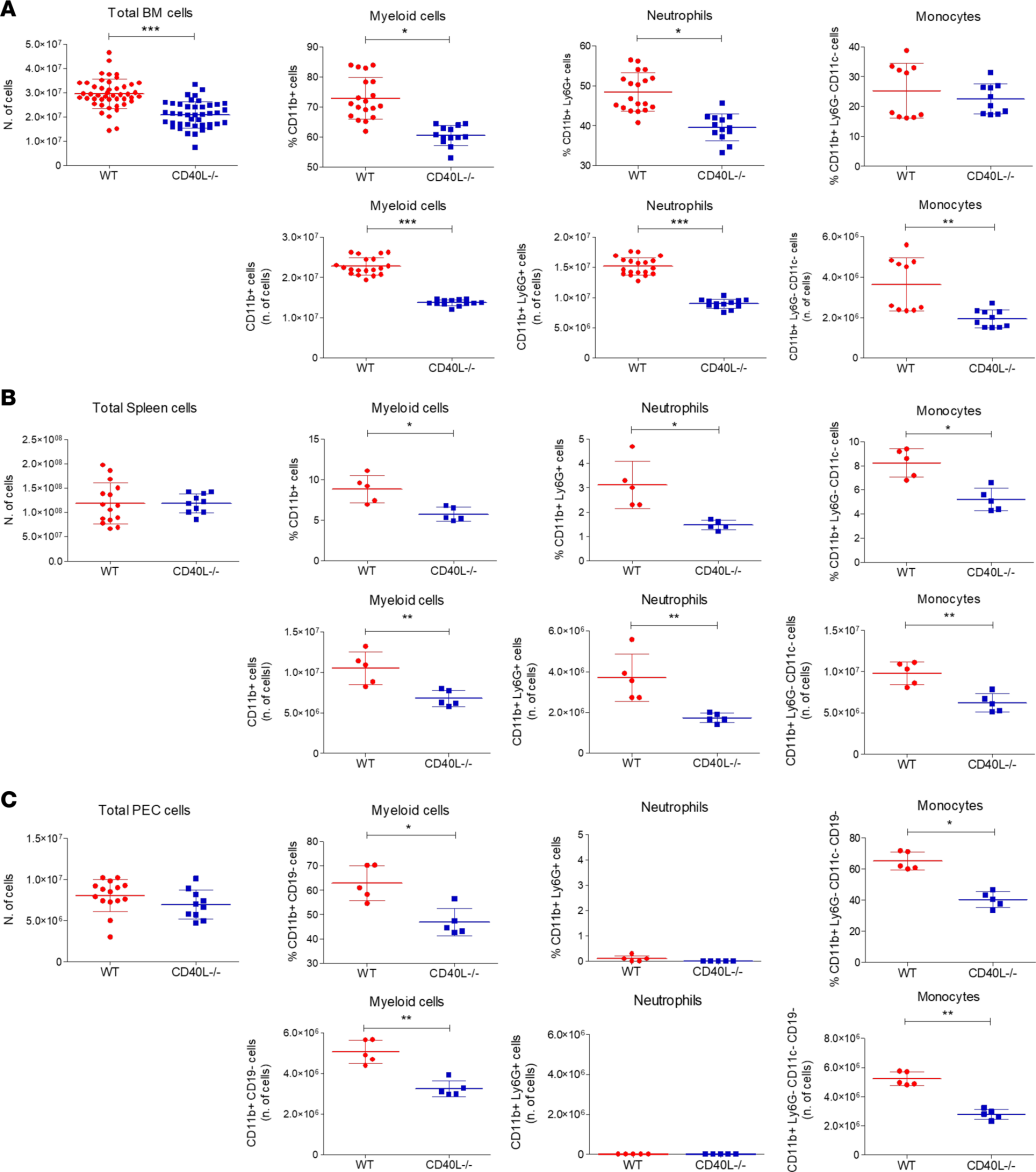
CD40L promotes myelopoiesis and granulopoiesis in vivo. (**A**) Total BM leukocytes from wild-type (WT) and CD40L knockout (CD40L^–/–^) mice (WT: *n* = 46; CD40L^–/–^: *n* = 42) and number/percentage of myeloid (CD11b^+^ cells), neutrophil (CD11b^+^ Ly6G^+^ cells), and monocyte (CD11b^+^ Ly6G^–^ CD11c^–^ cells) populations (WT: *n* = 19; CD40L^–/–^: *n* = 13; WT and CD40L^–/–^ monocytes: *n* = 10). (**B**) Total number of spleen leukocytes (WT: *n* = 15; CD40L^–/–^: *n* = 10), and number/percentage of myeloid, neutrophil, and monocyte populations (*n* = 5). (**C**) Total number of peritoneal exudate cells (PEC) (WT: *n* = 15; CD40L^–/–^: *n* = 10) and number/percentage of myeloid (CD11b^+^CD19^–^), neutrophil, and monocyte (CD11b^+^Ly6G^–^CD11c^–^CD19^–^) populations (*n* = 5). The number of subpopulations of each organ was obtained based on the percentage of cells compared with the cell number average obtained from 19 WT and 13 KO mice. *P < 0.05, **P < 0.01, ***P < 0.0001 (unpaired *t* test).****

To better understand the role of CD40L in leukocyte development, we compared the levels of myeloid and lymphoid cells in the BM of CD40L-deficient (CD40L-KO mice [CD40L^–/–^ or KO mice]) and WT female mice. We found that CD40L^–/–^ mice showed a significant reduction in the total number of BM cells when compared with the WT group (Figure 1A). The investigation of BM subpopulations revealed a reduction in the number and percentage of total myeloid cells (CD11b^+^), neutrophils (CD11b^+^Ly6G^+^), and monocytes (CD11b^+^Ly6G^–^CD11c^–^) (Figure 1A). Of note, the number of total myeloid cells in the KO group was equivalent to the number of neutrophils in the WT group (Supplemental Figure 1, A and B; supplemental material available online with this article; https://doi.org/10.1172/jci.insight.148652DS1). In turn, the percentage of lymphoid cells (T, B, and NK cells) in the BM was similar between groups; however, the absolute number of these cell subpopulations was reduced (Supplemental Figure 2A), suggesting a global hematopoiesis abnormality in the absence of CD40L that may affect all leukocyte subpopulations.

We then investigated the leukocyte distribution in peripheral organs. CD40L^–/–^ mice exhibited reduced quantities of both myeloid subpopulations (neutrophils and monocytes). We observed reduced levels of myeloid cells in both spleen and peritoneal cavity of CD40L^–/–^ mice compared with WT mice, while the total splenocyte count was similar between the groups (Figure 1, B and C). In the peritoneal cavity, this observation was specifically due to the reduced number of monocytes, since neutrophils are not present in the peritoneal cavity of normal mice under steady-state conditions (42). In terms of lymphoid cell subpopulations (B cells, T cells, and NK cells), although we found no alteration in the peritoneal cavity, spleens from CD40L^–/–^ mice displayed increased percentages and numbers of B cells associated with reduced numbers and percentages of NK cells (Supplemental Figure 2, B and C). These results suggest that CD40L plays a pivotal systemic role during leukocyte development in the BM as well as in the distribution of neutrophils throughout the body, and they suggest that CD40L deficiency causes hematopoiesis dysfunction.

### CD40L modulates the transcriptional machinery for neutrophil mobility.

**Figure 2:**
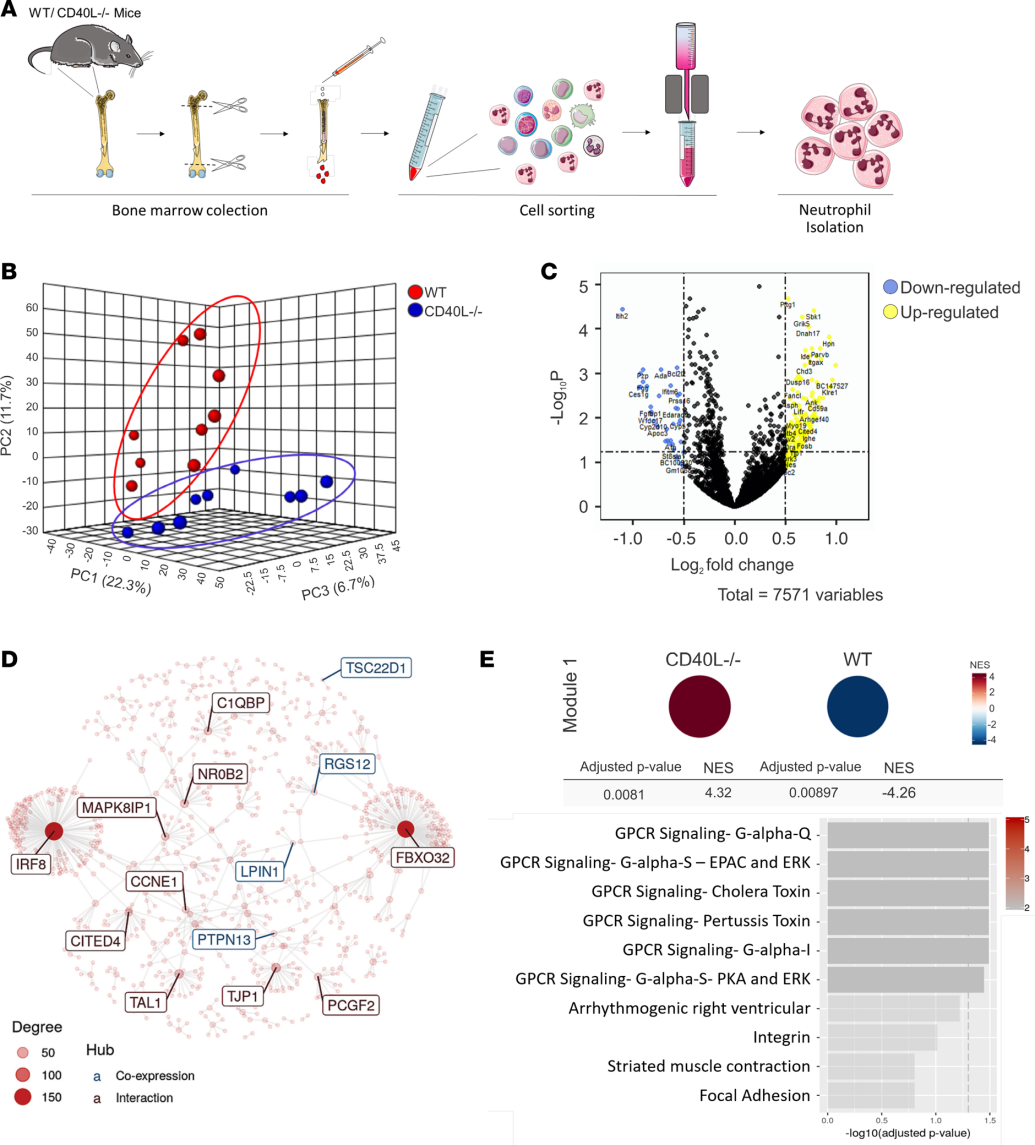
CD40L modulates the transcriptional profile of BM-derived neutrophils. (**A**) Schematic diagram of the neutrophil isolation protocol used for RNA isolation. (**B**) Principal component analysis (PCA) of transcriptome profile exhibited by CD40L^–/–^ and WT mice (*n* = 9/group). (**C**) Volcano plot representing the expression changes of all genes. Significantly down- and upregulated genes (adjusted *P* ≤ 0.05) are colored blue and yellow, respectively. Genes that do not show significant expression changes are colored black. Random labeling was performed in some genes of each side. (**D** and **E**) Modular gene coexpression analysis of all genes (figure showing the most enriched Module, called here as M1). Network interaction highlighting gene nodes with the potential hubs labeled (**D**) and gene set enrichment analysis shows the enrichment of Module 1 (symbol color represents the normalized enrichment score [NES]; top) and overrepresentation analysis of the enriched pathways in module 1 (−log_10_ adjusted *P* value, bottom; **E**).

**Figure 3:**
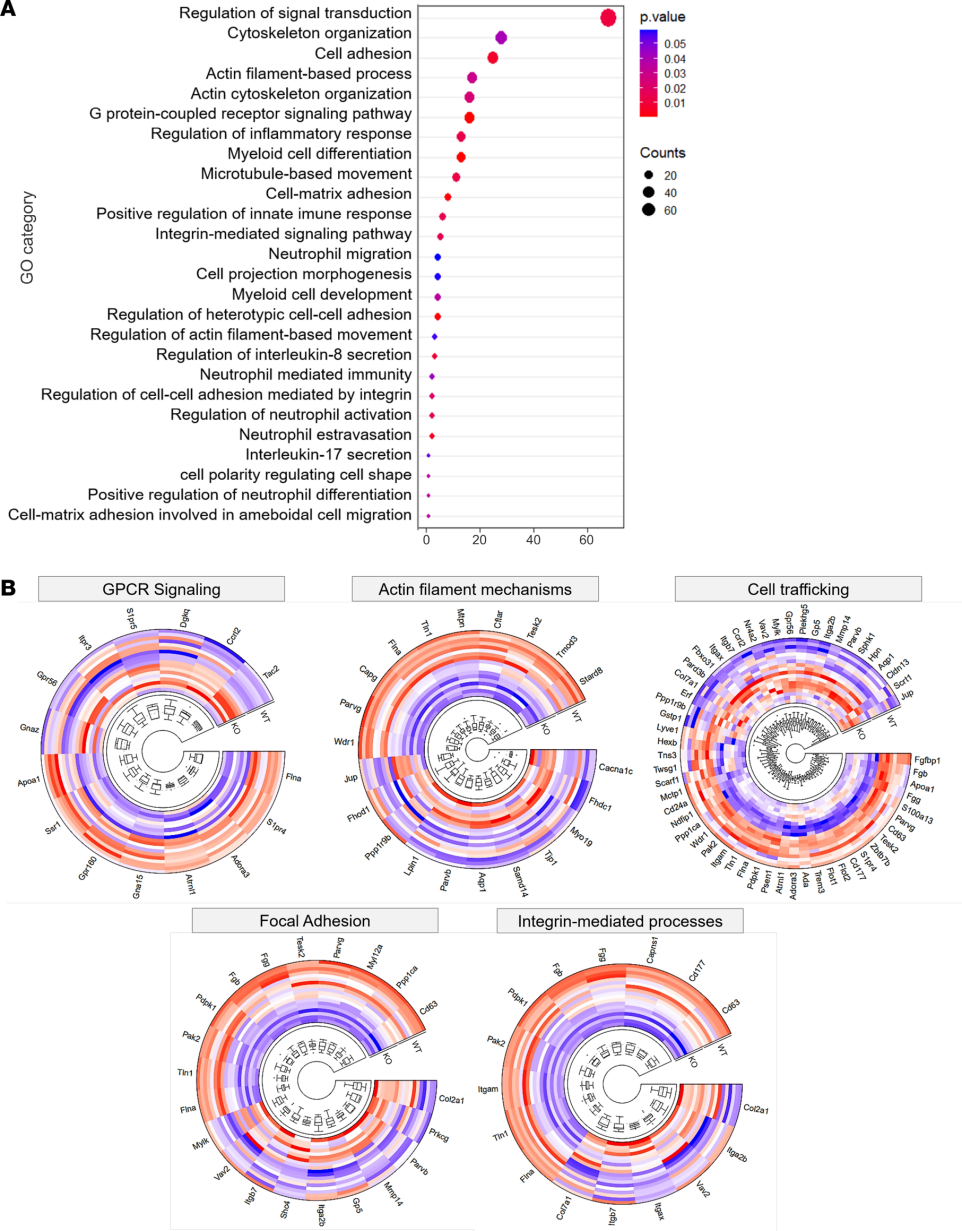
Gene ontology (GO) analysis of DEGs from neutrophils of CD40L–/– mice. (**A**) Biological processes predicted as affected by GO analysis (*P* ≤ 0.05). (**B**) Set of differentially expressed genes (DEGs) related to cell locomotion (GPCR signaling, actin filament mechanisms, cell trafficking, focal adhesion, and integrin-mediated processes).

Next, we sought to characterize the molecular mechanisms by which CD40L signaling regulates neutrophil development. Since neutropenia is a common hematological finding observed in CD40L-deficient patients (34, 35), and we recently reported that peripheral blood neutrophils have an immature phenotype (33), we performed high-throughput RNA sequencing (RNA-seq) of BM-isolated neutrophils from both WT and CD40L^–/–^ mice (Figure 2A). RNA-seq analysis revealed distinct transcriptome profiles when comparing WT and CD40L^–/–^ mice (Figure 2B). We identified 456 differentially expressed genes (DEGs; 219 upregulated and 237 downregulated) in CD40L^–/–^ mice when compared with WT (Figure 2C and Supplemental Data 1). In agreement with our findings showing abnormal neutrophil generation and distribution, modular gene coexpression analysis identified an enriched module of 380 genes (Figure 2D and Supplemental Data 2), containing genes involved in cell trafficking such as G protein-coupled receptor (GPCR) signaling, integrin, and focal adhesion (Figure 2E). These findings suggest that CD40L orchestrates in vivo the development of the transcriptional machinery for neutrophil mobility. In accordance, gene ontology (GO) analysis of DEGs indicated several dysregulated BPs related to cell trafficking and distribution throughout the body (Figure 3A and Supplemental Data 3), such as GPCR signaling, actin filament mechanisms, cell trafficking, focal adhesion, and integrin-mediated processes (Figure 3B).

**Figure 4:**
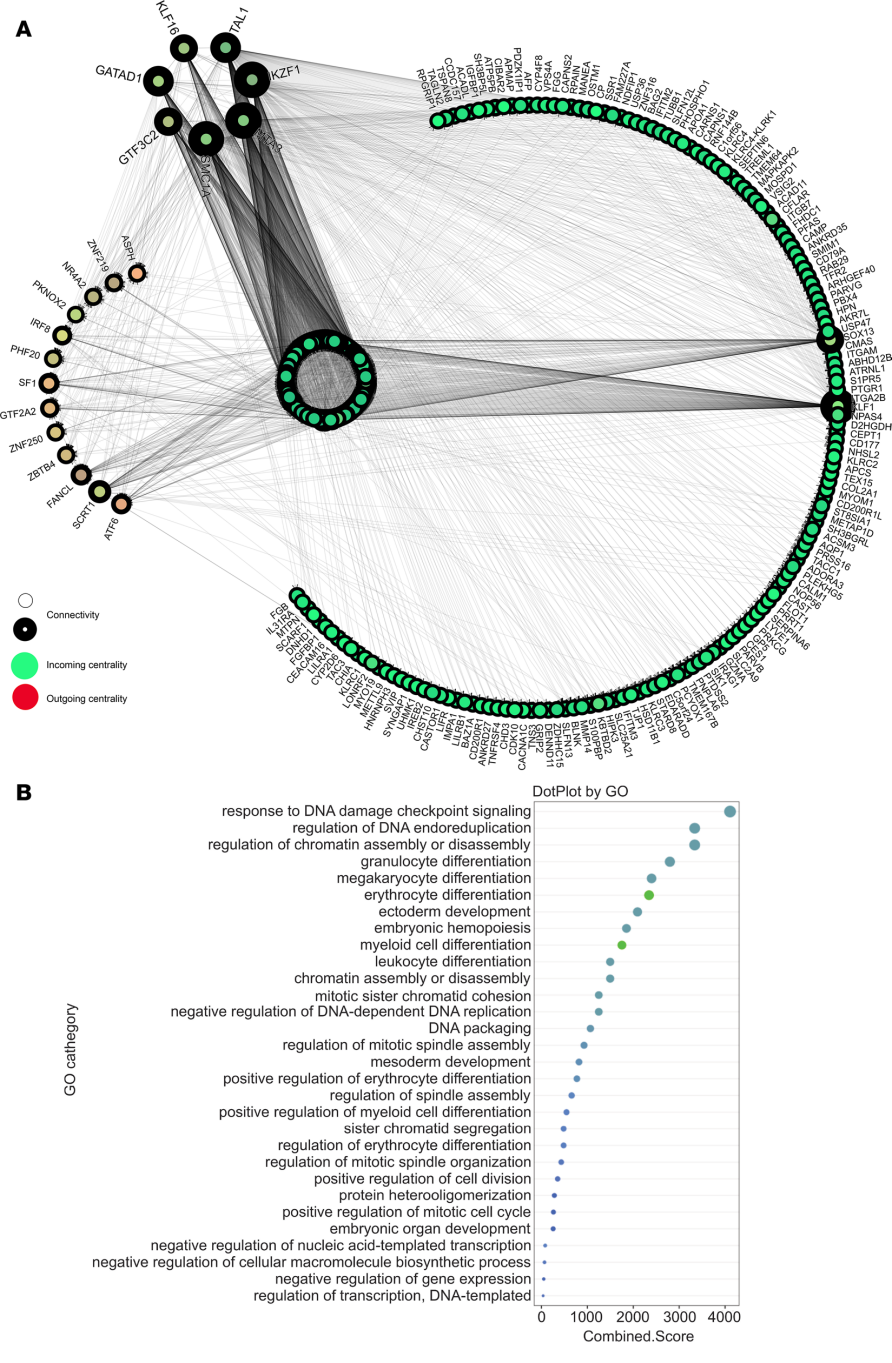
Transcriptional regulatory interactions involved in neutrophil development. (**A**) Network showing the interaction of transcription factors (TFs) differentially expressed by BM neutrophils from CD40L^–/–^ mice. All DEGs identified were used as input genes. Thickness of node outline corresponds to the connectivity (degree). Node color represents centrality based on direction: green is incoming, and red is outgoing, while the yellowish are mix of both. TFs are shown in the arc on the top; genes with ≥ 2 incoming centrality are highlighted in the arc on the bottom, and the remaining genes are organized in the circle in the middle (incoming centrality < 2). The network has 2671 edges (TF-gene connections). (**B**) The dot plot shows the results of enrichment analysis of top 10 TFs with highest connectivity (hubs).

Furthermore, several studies have demonstrated the relevance of transcription factors (TFs) in the regulation of neutrophil differentiation (43, 44). In line with these findings, we performed a TF–target gene analysis and identified a network of TF-target regulatory relationships among the DEGs of BM neutrophils from CD40L^–/–^ mice (Figure 4A). GO analysis of the top 10 TFs exhibiting the highest connectivity degree (hubs) revealed that they enrich BPs such as myeloid cell differentiation, granulocyte differentiation, and several cell cycle–associated processes (Figure 4B), indicating that the absence of CD40L results in key changes in a network of TF and genes involved in neutrophil development

### CD40L controls the dynamics of transcriptional regulation during neutrophil maturation.

Previous reports showed a maturation arrest of the neutrophil lineage at the promyelocyte-myelocyte stage in the BM of CD40L-deficient patients (34, 45), thus reinforcing the role of CD40L in neutrophil development. We further investigated this process by comparing the transcriptome exhibited by neutrophils from CD40L^–/–^ mice with publicly available gene expression data (NCBI’s Gene Expression Omnibus database; GEO GSE137538) generated by Xie and colleagues (39), which characterized the transcriptional profile of murine neutrophil developmental stages. First, we determined the DEGs observed in normal neutrophils at consecutive stages by comparing each developmental stage with its previous one. This enabled us to identify the transcriptomic signature of each differentiation stage. We obtained 528 DEGs for promyelocytes, 1382 for myelocytes, 785 for metamyelocytes, and 1252 for band-segmented neutrophils, the final maturation stage (Supplemental Figure 3, A and B, and Supplemental Data 4).

**Figure 5:**
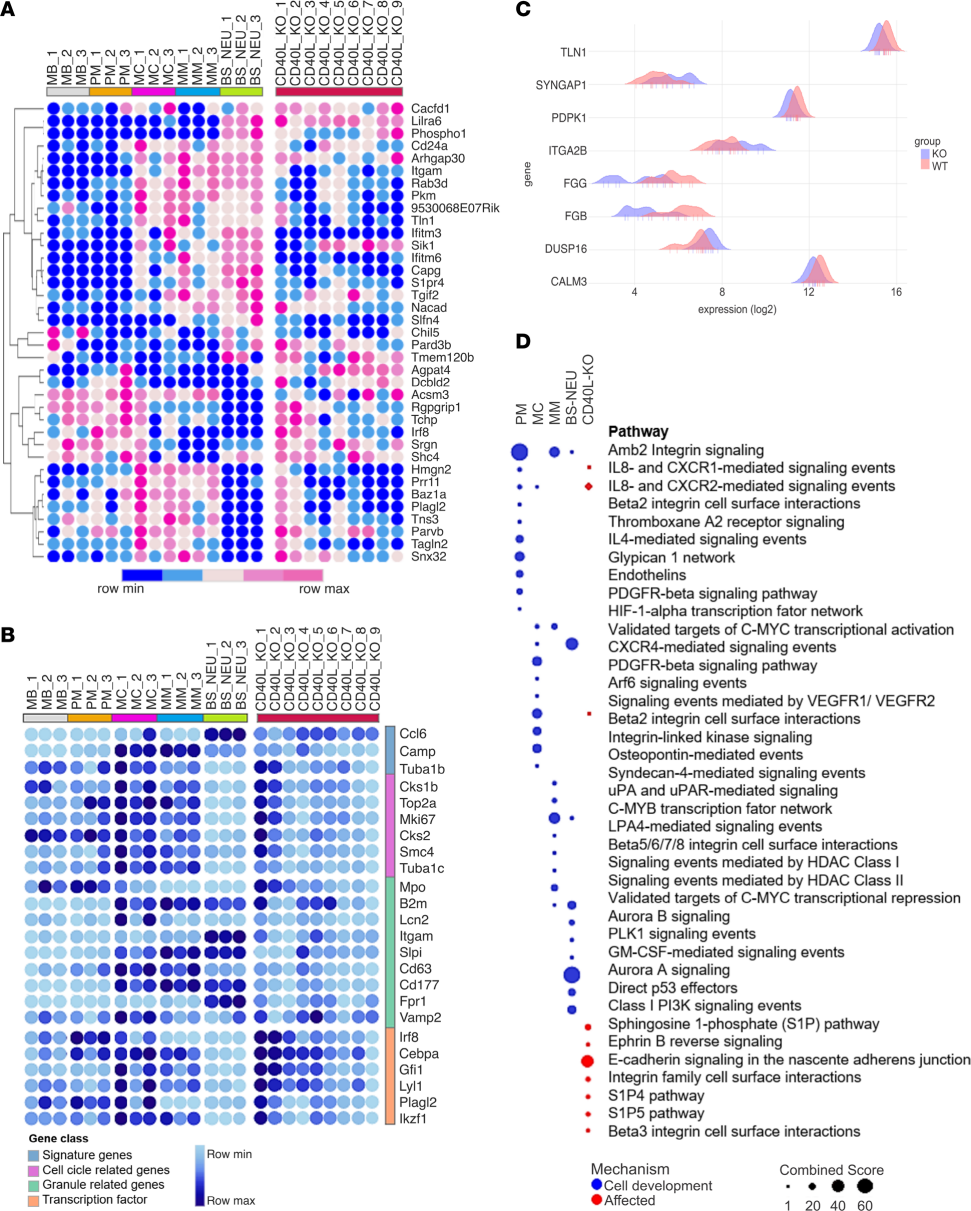
CD40L influences transcriptional dynamic during neutrophil maturation in BM. A publicly available data set was used to perform the following analysis (GSE137538). (**A** and **B**) Bubble heatmaps contrasting the pattern of expression of signature genes of each developmental stage obtained in a pairwise analysis compared with the DEGs presented by CD40L^–/–^ mice (**A**) or clustered in specific classes of genes (**B**) and compared with the transcription profile (DEGs and nonDEGs) exhibited by neutrophils from CD40L^–/–^ mice. (**C**) Histograms of DEGs involved in FLT3 signaling. (**D**) GO analysis of signature pathways of each neutrophil developmental stage (blue circles) and affected pathways identified in CD40L^–/–^ mice. MB, myeloblast; PM, promyelocytes; MC, myelocyte; MM, metamyelocytes; BS-neu, band/segmented neutrophil; CD40L_KO, CD40L^–/–^ mice.

Next, we compared the DEGs of each neutrophil developmental stage of WT mice with the DEGs of BM neutrophils from CD40L^–/–^ mice. The CD40L^–/–^ group showed more genes in common with the myelocyte stage (Supplemental Figure 3, C and D), which is in agreement with the maturation arrest reported in human CD40L deficiency (34, 45). However, neutrophils from CD40L^–/–^ mice also exhibited the transcriptional pattern overlapping with other stages of neutrophil development (Figure 5, A and B), suggesting that CD40L orchestrates the sequential development of neutrophils rather than interrupts the process at a specific stage. We also compared classes of genes specifically related to neutrophil development (39) and identified TFs associated with neutrophil development (Irf8, Plagl2, and Ikzf1; Figure 5D) (4, 8, 46–53) and genes involved in Flt3 signaling (Figure 5C), which is essential for myeloid cell development (9).

Finally, we performed a GO analysis of DEGs to compare enriched pathways of each developmental stage with those dysregulated in CD40L^–/–^ mice. We found that, in CD40L^–/–^ mice, the pathways that were affected included “IL8- and CXCR1-mediated signaling events,” “IL8- and CXCR2-mediated signaling events,” and “Beta 2 integrin cell surface interactions,” which are predicted to be acquired during neutrophil development at the promyelocyte-myelocyte stage (Figure 5D). Collectively, these findings indicate that CD40L modulates the transcriptional program that controls neutrophil maturation, and its absence impairs murine neutrophil development.

### CD40L plays a conserved role in both mouse and human neutrophil development.

**Figure 6:**
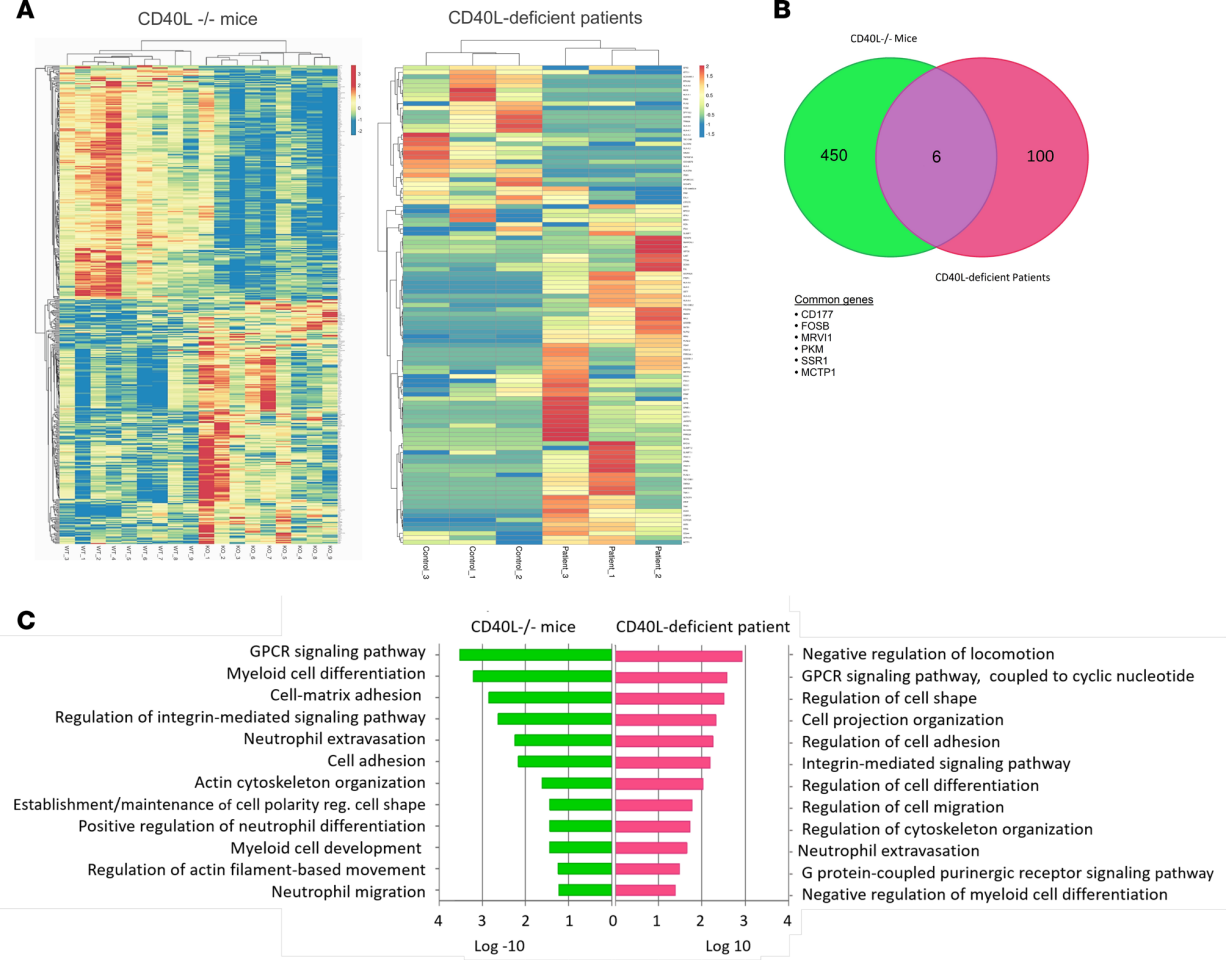
Conserved role of CD40L in neutrophil development in both mice and humans. The transcriptome of peripheral venous blood neutrophils from patients with CD40L deficiency was used to perform the analysis (Array Express database; data set E-MTAB-5316). (**A**) Heatmap of hierarchical clustering of the 456 DEGs identified in CD40L^–/–^ mice (mice) and 106 DEGs from CD40L-deficient patients (adjusted *P* ≤ 0.05). (**B**) Venn diagram showing the shared genes between CD40L^–/–^ mice and CD40L-deficient patients. (**C**) Biological processes commonly affected in both CD40L^–/–^ mice and CD40L-deficient patients were obtained from gene ontology (GO) analysis (*P* ≤ 0.05).

**Figure 7:**
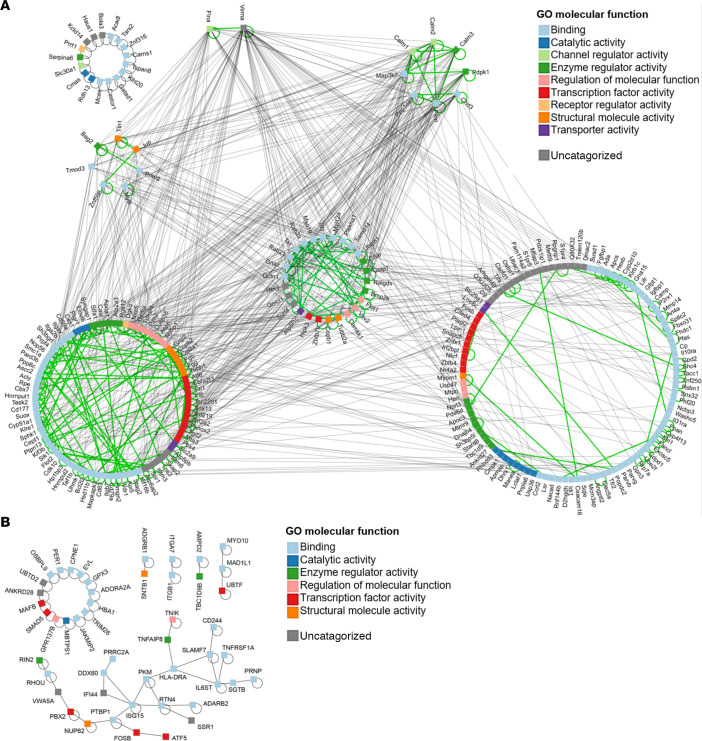
Common molecular functions and conserved physical protein interactions between mice and human DEGs. (**A** and **B**) Interaction network showing the conserved physical protein-protein interactions of DEGs and GO molecular function category of the genes from both CD40L^–/–^ mice (**A**) and CD40L-deficient patients (**B**). Gray lines represent the interactions between the genes products. Green lines highlight interactions among proteins with the similar number of interacting partners. For example, Flna and Virma have 35 and 69 interacting parters, respectively; proteins in the top right circle have 23–27 partners, while the bottom right circle includes proteins with only 1–3 partners. All interactions are listed in Supplemental Data 5.

Given the effect of CD40L on the development of neutrophils in the BM, we wondered if these effects are conserved in mice and humans. Since we had no access to BM from CD40L-deficient patients, we compared the transcriptome of CD40L^–/–^ mice with that of peripheral blood neutrophils from patients with CD40L deficiency (33) (Array Express database; data set E-MTAB-5316; https://www.ebi.ac.uk/arrayexpress/experiments/E-MTAB-5316/) (Figure 6A and Supplemental Data 1). Although we were able to identify only 6 common DEGs between mice and humans (CD177, FOSB, MRVI1, PKM, SSR1, and MCTP1; Figure 6B), we found common affected BPs related to cell development and trafficking in both species (Figure 6C). To determine the function of both mouse and human DEGs, we characterized relevant physical protein-protein interactions (PPIs) of each species. We first mapped all DEGs to their corresponding proteins using UniProt (Supplemental Data 5), identified all physical interactions using Interologous Interaction Database (IID; see below) (Supplemental Figure 4 and Supplemental Data 5), and then focused on interactions conserved between the 2 species and their molecular function (Supplemental Data 5). The analysis shows that, despite the lack of overlap between the DEGs identified in mice and humans, these genes share common molecular functions and are linked by conserved physical protein interactions (Figure 7, A and B). Altogether, these results suggest that CD40L modulates neutrophil development and distribution in both mice and humans, indicating a central role of this molecule in the fate of neutrophils.

### Neutrophils from patients with CD40L deficiency exhibit impaired migration in response to IL-8.

**Figure 8:**
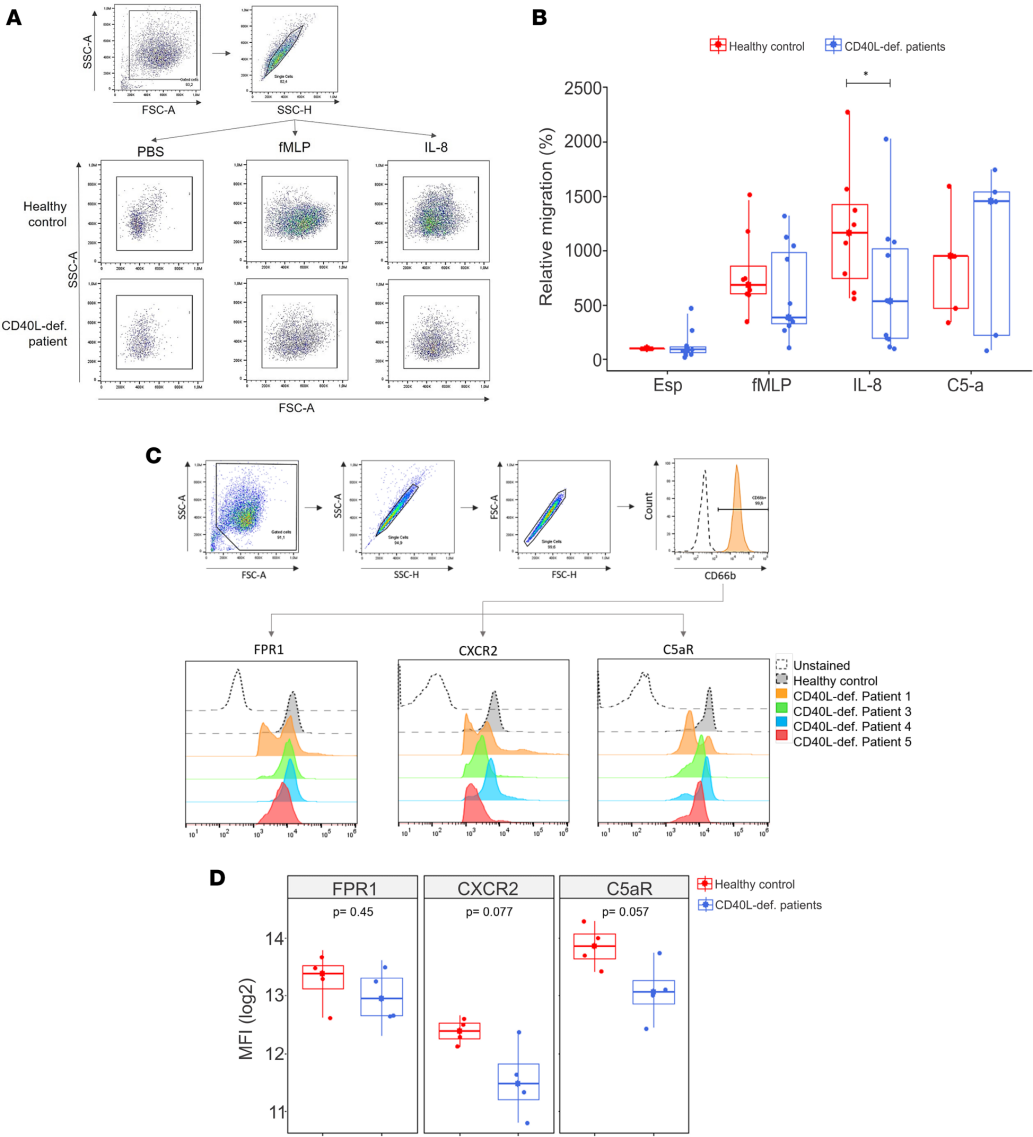
Neutrophils from patients with CD40L deficiency show impaired migration. (**A**) Representative figure of gating strategy used to quantify migrated neutrophils (*n* = 7). (**B**) Neutrophils from patients with CD40L deficiency and healthy individuals (healthy control) were incubated with PBS, fMLP (20 nM), IL-8 (10 nM), or C5a (25 nM). Spontaneous migration (PBS) of healthy individuals was normalized to 100%, and the other conditions were compared with the normalized value to assess the relative migration number (expressed in %) (*n* = 7 [P1, P2, and P3 evaluated more than once]; C5a: *n* = 5). (**C**) Gating strategy and histograms showing the pattern of expression of FPR1, CXCR2, and C5aR receptors in CD66b^+^ neutrophils from patients with CD40L deficiency (colored) and healthy individuals (gray, dotted line). (**D**) Expression of FPR1, CXCR2, and C5aR receptors expressed in log_2_ obtained from median fluorescence intensity (MFI) values. *n* = 4. *P < 0.05 (Mann-Whitney *U* test).

Next, we asked whether the results obtained in the transcriptome analysis were predictive of biological effects on human neutrophils. We enrolled 7 patients with CD40L deficiency in our study (clinical history and mutations in CD40L are summarized in Table 1). In agreement with previous works that reported the reduction in neutrophil migration in response to inflammatory stimuli in CD40^–/–^ (54) and CD40L^–/–^ mice (33), neutrophils from CD40L-deficient patients showed reduced migratory capacity in response to IL-8 when compared with healthy subjects (Figure 8, A and B), reinforcing the prediction of defective IL-8/CXCR2–mediated signaling (Figure 5D).

Finally, we found that neutrophils from CD40L-deficient patients presented a tendency of reduced CXCR2 (IL-8 receptor) and C5aR (C5a receptor) expression, while the expression of FPR1 (fMLP receptor) varied among patients between normal and reduced (Figure 8, C and D). However, the comparison between the migration response and the level of receptor expression did not indicate that the reduced migration toward the chemoattractant is a consequence of reduced expression of its receptor (data not shown). We also evaluated actin polymerization of neutrophils by flow cytometry (data not shown), but we found no statistical difference when comparing healthy subjects and CD40L-deficient patients, suggesting that the change in migratory capacity is not directly or exclusively related to impaired receptor expression and could be the result of intrinsic neutrophil defects as a consequence of an abnormal development in the BM.

## Discussion

**Figure 9:**
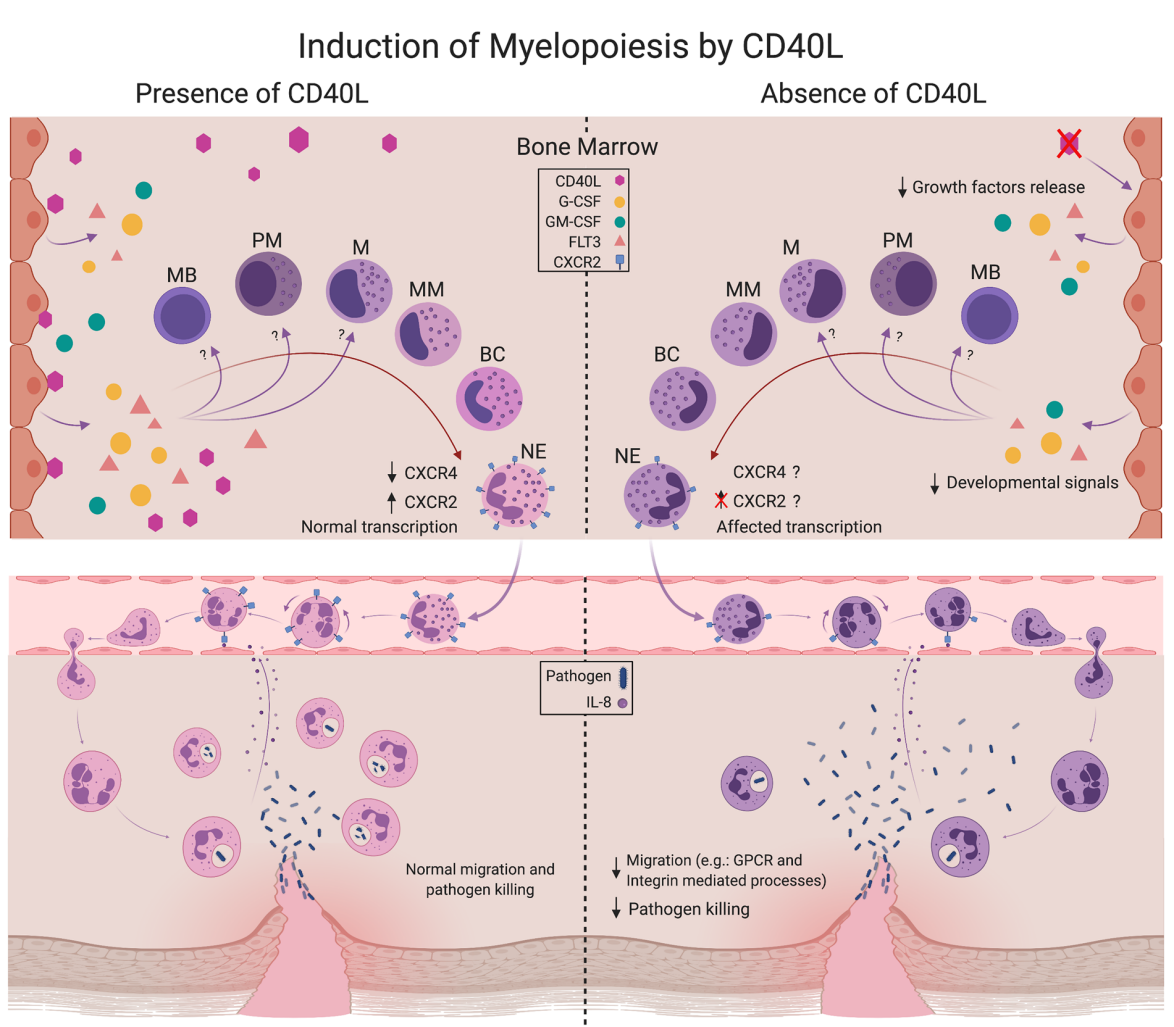
Proposed mechanism of CD40L-induced myelopoiesis. Our results suggest that CD40L orchestrates neutrophil development and trafficking by modulating transcriptional signatures in the BM. The presence of CD40L induces BM stromal cells to release growth factors (e.g., G-CSF, GM-CSF, and flt^3^L; refs. 22, 23), which act on myeloid lineages (specifically neutrophils), influencing the production and development of these cells (9, 21). Mature neutrophils are released into the bloodstream with the capacity to migrate to sites of infection in response to chemoattractants and kill pathogens (2). In the absence of CD40L signaling, the generation and development of the myeloid lineage in the BM is impaired and, more specifically, results in the generation of neutrophils with dysregulated transcriptome profile that affects the ability to traffic throughout the body, migrate to sites of infection in response to inflammatory signals, and properly eliminate invading pathogens (33). MB, Myeloblast; PM, promyelocytes; MC, myelocyte; MM, metamyelocytes; BC, band cell; NE, neutrophil.

Our integrative approach indicates that CD40L orchestrates transcriptional signatures of BM neutrophils that associate with development and traffic. Both mouse and human data suggest a nonredundant and conserved role of CD40L in granulopoiesis, promoting the generation of mature neutrophils able to reach the circulation and peripheral organs (Figure 9). The reduction in the number of neutrophils in the BM and spleen of CD40L^–/–^ mice and the transcriptomic analysis reinforce the hypothesis of developmental defects generated by lack of CD40L signaling. Thus, our work provides insights into the influence of CD40L on cell development and mechanisms that regulate myelopoiesis.

Our data agree with previous reports demonstrating the role of CD40L in innate immune cells, such as DCs (31), macrophages (32), and neutrophils (33). Ref. 33 reports that peripheral blood neutrophils from CD40L-deficient patients exhibit an immature phenotype associated with defective effector function, such as a decreased microbicidal activity. Moreover, patients with CD40L deficiency exhibit an arrest of myeloid lineage maturation in the BM at the promyelocyte-myelocyte stage (34, 45), although the causative mechanism remains unknown. Our findings highlight the pleiotropic role of CD40L and indicate that CD40L signaling may be essential to neutrophil development, and they show that its absence results in wide-ranging consequences for the innate immune response and the maintenance of homeostasis.

High-throughput transcriptomics has been shown to be a powerful tool to study intrinsic changes at the molecular level and cellular perturbation in various conditions (55). Transcriptome analysis of neutrophils from CD40L-deficient mice and patients revealed an altered transcriptional profile compared with neutrophils from WT mice and healthy subjects, respectively. Both models showed that the absence of CD40L affects BPs related to cell development and neutrophil trafficking, despite the differences of species and neutrophil source (peripheral blood versus BM). In our study, BM neutrophils from CD40L^–/–^ mice showed altered expression of TFs highly interconnected with genes involved in myelopoiesis (4, 8, 46–53), thus suggesting that CD40L modulates a transcriptional regulatory network of molecules involved in neutrophil development. Moreover, our results indicate dysregulation of diverse genes coding for proteins involved in FLT3 signaling, an important regulator of hematopoiesis, which is in accordance with a previous study demonstrating that CD40L stimulates myelopoiesis by regulating FLT3L production in BM stromal cells (22).

In agreement with these data, CD40L^–/–^ mice showed a significant reduction in the total number of nucleated cells in the BM, namely in the number and percentage of the myeloid population as a consequence of a reduction in the granulocytic lineage. This observation is in agreement with previous reports describing a maturation arrest of the myeloid lineage at the promyelocyte-myelocyte stage in the BM of CD40L-deficient patients (34, 45). Together, these data suggest that the reduction of myeloid cells in the periphery of CD40L-deficient patients and mice is due to a defective myeloid production rather than an impaired release and migration from the BM. Therefore, the dysregulated genes detected by RNA-seq, such as those related to migration and trafficking, are possibly due to defective neutrophil development.

Several investigators have also reported the influence of sCD40L-CD40 signaling on inflammation, homeostasis, and pathological conditions (56, 57). In nonhematopoietic cells, such as endothelial cells, fibroblasts, and epithelial cells, CD40L-CD40 signaling is involved in the amplification and regulation of inflammatory responses by inducing the expression of adhesion molecules (E-selectin, VCAM, and ICAM) and the secretion of proinflammatory cytokines (58–60). Moreover, several studies described the effect of sCD40L on neutrophil activation by its binding to CD40 (61) or interaction with MAC-1, a key molecule for neutrophil recruitment to the site of infection (62–65). Our group and others have demonstrated in vivo that CD40L^–/–^ mice show a reduction in the recruitment of neutrophils into the peritoneal cavity in response to stimulation with thioglycollate (33, 54) or infection by *Salmonella typhimurium* (33), indicating a failure in trafficking and distribution of neutrophils to inflammatory sites. In agreement, our transcriptomic approach suggests a broad role of CD40L in the regulation of neutrophil distribution by regulating BPs such as GPCR signaling, cell trafficking, and integrin-mediated pathways in the periphery and during neutrophil development.

Furthermore, in agreement with our mouse model, neutrophils from CD40L-deficient patients showed a reduction in IL-8–induced migration. Surprisingly, no clear relationship between reduced migration toward IL-8 and reduced expression of its receptor was found, although this could be attributed to the limited number of patients analyzed. However, considering the wide range of neutrophil transcriptomic alterations, the main hypothesis is that CD40L modulates neutrophil migration in a systemic manner rather than by controlling the expression of chemoattractant receptors. Nonetheless, our manuscript has several limitations that need to be addressed by future mechanistic studies. For instance, while CD40L has been shown to be a central player in B lymphocyte survival (66) and exert pleiotropic homeostatic roles during granulopoiesis (23), the impact of its absence in neutrophil survival remains to explored. Thus, in the absence of this molecule, it is possible that myeloid progenitors undergo a higher rate of apoptosis in the BM and/or have a delayed transit time through this primary lymphoid organ, impeding their development. Although we did not observe a difference in the viability of BM cells isolated from normal or CD40L-deficient mice (data not shown), this possibility remains to be investigated.

Recombinant human G-CSF (rhG-CSF) has been used for the treatment of neutropenia in CD40L deficiency (67). However, although the number of peripheral neutrophils can reach normal levels under rhG-CSF treatment (68, 69), we have recently reported that neutrophils from CD40L-deficient patients, even when receiving rhG-CSF, present functional defects. This fact indicates that the replacement of this cytokine alone is not enough to recapitulate normal myeloid development. In this context, it has been previously shown that the CD40L-CD40 interaction orchestrates the myeloid cell development directly by activating myeloid cell progenitors and indirectly by modulating the production of growth factors such as GM-CSF, G-CSF, thrombopoietin, and FLT3L in the BM (19, 22–24). Thus, it is possible that a defective production of any of these growth factors might be involved in the impaired neutrophil development observed in the absence of CD40L in both humans and mice. This possibility could also be implicated in the etiology of neutropenia presented by patients with a variety of primary T cell defects and/or those infected with HIV (70), and these hypotheses remain to be explored. If that is the case, replacement with G-CSF, GM-CSF, and/or FLT3L could be investigated to restore the normal myeloid development.

Altogether, these findings indicate that the modulation of myeloid cell development by CD40L is a complex process that involves a network of several cells and signaling pathways. In this context, although it was originally thought that CD40L influenced the inflammatory response by interacting only with its classic binding partner (CD40), at least 3 other CD40L receptors — the integrin αIIβ3 expressed on platelets, α5β1 on endothelial cells, and αMβ2 (CD11b or MAC-1) on myeloid cells (24, 62, 71, 72) — might also participate. Thus, future studies will need to investigate the individual or synergistic involvement of these CD40L receptors in the development of myeloid cells.

In conclusion, our work suggests that the CD40-CD40L interaction may play an important regulatory role in the development of neutrophils in the BM by orchestrating a network of molecules involved in cell trafficking throughout the body and migration to sites of infection in response to inflammatory signals, providing insights into the complex relationships between CD40L signaling and myelopoiesis.

## Methods

### Animals.

C57BL/6 WT mice were purchased from the Jackson Laboratory. CD40L-deficient mice were provided by Richard Flavell (Department of Immunobiology, Yale University School of Medicine, New Haven, Connecticut, USA) and have been previously described (73). Mice were bred at the animal facility in the College of Medicine and Health Sciences (UAE University), received rodent chow and water ad libitum. For all procedures, adult female mice from both strains were matched by age (8–12 weeks of age) and weight.

### Organs harvesting and processing.

Groups of mice (5 mice per group) were euthanized by inhalant anesthetic isoflurane (Zoetis) according to institutional guidelines (College of Medicine and Health Sciences, UAE University.). Processing of cells from the spleen and peritoneal cavity was done as previously detailed (74, 75). BM cells were harvested aseptically from both femur and tibia bones and processed immediately for analysis. Cell count and viability were determined by Trypan blue exclusion.

### Multiparametric flow cytometry.

Processed cells were stained for flow cytometry analysis, as previously described (76). Briefly, isolated cells were washed in PBS, resuspended in staining buffer (PBS/1% FCS/0.1% NaN_3_) and aliquots of 100 μL were dispensed into the wells of a 96-well round-bottom plate. Cells were incubated with anti-CD16/CD32–specific mAb (101320/93, BioLegend) for 30 minutes on ice to block FcγRΙΙΙ/ΙΙ receptors. The cells were then stained with 7-AAD viability dye (420404, BioLegend) to gate live cells. Cells were then stained with a panel of antibodies to lymphoid or myeloid cell surface markers (BioLegend) for 30 minutes on ice. The lymphoid panel included CD3-FITC (catalog/clone 100204/17A2), CD4-APC (catalog/clone 116014/RM4-4), CD8-APC-Cy7 (catalog/clone 100714/53-6.7), and CD19- PE-Dazzle 594 (catalog/clone 115554/6D5) antibodies. The myeloid panel included CD11b–Alexa Fluor 488 (catalog/clone 101217/M1-70), Ly6G-BV605 (catalog/clone 127639/1A8), NK1.1–PE-Dazzle 594 (catalog/clone 108748/PK136), CD11c–Alexa Fluor 700 (catalog/clone 117320/N418), and MHC class II–APC-Cy7 (catalog/clone 107628/M5/114.15.2). All antibodies were pretitrated in preliminary experiments and used at saturating concentrations. After washing, cells were analyzed on a BD FACSCanto II (BD Biosciences) and analyzed using BD FACSDiva software (BD Biosciences).

### Mouse neutrophil isolation.

Mouse BM neutrophils were purified by negative selection on an autoMACS cell separator using a neutrophil isolation kit (Miltenyi Biotec) and following the manufacturer’s instructions. The cell purity obtained using this procedure was verified by FACS staining of purified cells using antibodies specific to CD11b and Ly6G and was routinely found to be 90%–95%.

### RNA-seq.

After purification, RNA from BM neutrophils was isolated by TRIZOL reagent (Invitrogen) according to the manufacturer’s instructions. When necessary, cells obtained from 2 different mice of the same group and processed simultaneously were pooled to obtain the number of cells required to perform the sequencing. RNA integrity and concentration were assessed using the Agilent 2100 Bioanalyzer RNA Nanochip. Samples with RNA integrity number (RIN) ≥ 8 were used for the transcriptome analysis. cDNA libraries were obtained using the Illumina CBot station, and HiScanSQ was performed using the NEBNext Ultra Sample Preparation Kit (Illumina Inc.) according to each manufacturer’s instructions. Sequencing was carried out using the Illumina HiSeq 4000 platform (150-nucleotide paired-end reads).

### Bioinformatics analysis.

After quality assessment, reads were aligned to the reference genome using STAR software, and the gene expression level was estimated after data normalization by using the method fragments per kilobase of transcript sequence per million (FPKM) base pairs sequenced. Differential expression analysis of the groups was performed using DESeq2 through the NetworkAnalyst platform (77). For data visualization, we used different bioinformatics tools. A volcano plot graph was generated using VolcanoPlot R package, coexpression analysis was performed using WebCemiTool (78), Circular Heatmaps were generated using Circlize R package, Circleplot was built using Circos Table Viewer (79), heatmaps were generated using ClustVis (80), bubble heatmaps were generated using Morpheus (https://software.broadinstitute.org/morpheus/), and Venn diagrams were created using InteractiVenn (81). GO analyses were performed using Panther (82) and NCI-Nature (83), and pathway interactions were created via the Enrichr platform (84). Physical PPIs for both mice and humans were obtained from Interologous Interaction Database (IID v2020-05) (85). Protein interactions (Supplemental Data 5) were analyzed, and figures were prepared using NAViGaTOR v3.0.14 (86). SVG output from NAViGaTOR was finalized in Adobe Illustrator 2021 to include legends, and the resulting 300DPI PNG file was submitted. Proteins were annotated with GO molecular function (obtained from UniProt).

### Transcriptional regulatory interactions.

We used Catrin (Catalogue of Transcriptional Regulatory Interaction; Version 1.1.0.1; Database version 1.1.0.3; http://ophid.utoronto.ca/Catrin/) to analyze the transcriptional regulatory interactions involved in neutrophil development. Catrin integrates data obtained from 15 independent resources, including TF–target gene associations derived from ChIP high-throughput experiments, gene regulatory network inference algorithms, and machine learning tools. Network analysis and visualization was performed using NAViGaTOR v 3.0.14. The final figure with legend was prepared in Adobe Illustrator 2021 from the exported SVG file.

### Public data set selection.

Publicly available RNA-seq data sets were obtained from the public functional genomics data repository Gene Expression Omnibus (GEO) DataSets (87, 88) and ArrayExpress (89). The terms “neutrophil” and “development” were used as search terms. To select data as similar as possible to the data set generated in our study, the data sets were filtered based on organism (*Mus musculus)*, neutrophil source (BM neutrophils), and type of study (expression profiling by high throughput sequencing). As quality control criteria, we excluded all data sets with less than 3 samples available for each subpopulation and data sets lacking information about the experimental design and with no publication available. Finally, among the remaining studies, we selected the data set in which the authors correlated the subpopulations of neutrophils identified in the study to the classic different stages of development of neutrophils (myeloblasts, promyelocytes, myelocytes, metamyelocytes, and mature cells). One data set matched to the filters applied and was used in the study (GSE137538) (39). For the human model, only 1 data set was available matching the terms “neutrophils” and “CD40L-deficient patients” (E-MTAB-5316).

### Human subjects.

Samples from 8 unrelated patients with CD40L deficiency were collected to perform the experiments. The summary of the patients’ clinical history and genetic characteristics are described in Table 1. For each experiment, a healthy subject was included for comparison. Peripheral blood samples were collected under institutional guidelines (Institute of Biomedical Sciences, University of São Paulo).

### Human neutrophil isolation.

Neutrophils from CD40L-deficient patients and healthy subjects were obtained from heparinized peripheral blood by Ficoll-Hypaque (GE Healthcare) isolation. Briefly, 10–20 mL of blood were collected in a sterile tube with sodium heparin, diluted in 10–20 mL of 6% Dextran, and incubated for 20 minutes at 37°C. The fraction rich in leukocytes (upper layer) was transferred to a tube containing 12 mL of Ficoll-Hypaque (density 1.077 g/mL) and centrifuged for 20 minutes at 900*g* at room temperature without braking. After centrifugation, the supernatant was discarded and the remaining red cells were lysed by adding 3 mL of cold sterile distilled water followed by the addition of 6 mL of PBS and centrifugation (10 minutes at 600*g* at room temperature). The cell pellet with polymorphonuclear leukocytes was suspended in RPMI 1640 medium (Invitrogen). Cell viability was consistently > 96%, as determined by Trypan blue exclusion.

### Cell migration assay.

Neutrophil migration in response to chemoattractive factors was evaluated by using a 24-well plate–containing chambers with a 5 μm pore-permeable polycarbonate membrane (Corning). Briefly, neutrophils (5 × 10^5^ cells) were suspended in 200 μL PBS, added to the upper chamber (transwell), and incubated for 30 minutes at 37°C for cell sedimentation. Subsequently, the chambers were transferred to wells containing 500 μL of PBS or PBS with the chemoattractant fMLP (20 nM), IL-8 (10 nM), or C5a (25 nM). The plate was incubated for 45 minutes at 37°C for cell migration from the upper chambers toward the lower wells. Following incubation time, the suspension of the lower wells was collected and transferred to 5 mL round-bottom tubes for cytometry (Corning). For the dissociation of cells adhered to the wells, 300 μL of PBS with 2 mM EDTA was added in each well, and the plate was incubated for 15 minutes at 37°C. Cells were fixed with 0.5% PBS-paraformaldehyde, and the number of cells that migrated was quantified using a flow cytometer (Attune NxT; Thermo Fisher Scientific). The spontaneous migration presented by the healthy subjects was normalized to 100%; then, the other conditions were compared with the normalized value and expressed as relative migration. The migration of the patient’s neutrophils was compared with the migration showed by the healthy subjects processed concomitantly to exclude possible variations due to different processing day and conditions.

### Expression of chemokine receptors.

The expression of C5aR, fMLP-R, and CXCR2 receptors was quantified by flow cytometry. Before staining, neutrophils (2 × 10^5^) were incubated with blocking buffer (PBS with 1% human IgG and 2% FBS) for 10 minutes to block Fc-receptors and avoid nonspecific binding. The cells were suspended in 50 μL PBS for staining with anti–CD66b-PE (561650/G10F5; BD Biosciences), –C5aRr-PerCP/Cy5.5 (344312/S5-1), –fMLP-R-APC (391610/W15086B), and –CXCR2-APC/Fire750 (320720/5E8) antibodies (all from BioLegend) for 30 minutes at 4°C in the dark. After incubation time, the cells were washed twice with PBS and fixed in PBS-paraformaldehyde 0.5%. The analysis was performed by flow cytometry (Atune NxT). The neutrophil population was selected based on cell size versus cell complexity (forward scatter [FSC] versus side scatter [SSC]), and the chemokine receptor expression was analyzed in the CD66b^+^ population (a specific marker of human neutrophils). The analysis was performed using the FlowJo software vX.0.7 (BD Biosciences).

### Data availability.

All data that support the findings of this study are available within the paper and its supplemental material or are available from the corresponding author upon reasonable request. Publicly available data sets used in this study were GSE137538 available at “NCBI GEO datasets” (https://www.ncbi.nlm.nih.gov/gds) and E-MTAB-5316 available at “Array Express” (https://www.ebi.ac.uk/arrayexpress/). RNA-seq data from C57BL/6 WT and CD40L-KO mice are available in the ArrayExpress database (http://www.ebi.ac.uk/arrayexpress) under accession number E-MTAB-10732.

### Statistics.

Statistical significance was assessed by nonparametric tests. Data were expressed as median ± SD with 25th and 75th percentiles, and with mean ± SD. The statistical analyses were performed using the GraphPad PRISM 5.01 software (GraphPad Software), and differences with a *P* ≤ 0.05 were considered significant. Statistical significance was assessed by using the 2-tailed, unpaired *t* test for parametric samples. Statistical significance was assessed by using the Mann-Whitney *U* test for nonparametric samples.

### Study approval.

All studies involving animals were carried out following and after approval of the animal research ethics committee of the College of Medicine and Health Sciences, UAE University. Written informed consent was received from all the participants, healthy subjects, and patients or their parents, prior to inclusion in the study. The study approval was obtained from the Ethics Committee of the Institute of Biomedical Sciences, University of São Paulo.

## Author contributions

TTF, BKAR, ACN, and OCM conceived the study and designed the experiments; TTF, AAS, GB, and YAM performed experiments; TTF, AAS, and BKAR analyzed experimental data; TTF, SMDSN, RCS, IJ, and OCM performed computational analysis; TTF and OCM wrote the manuscript draft; LAB, BKAR, ACN, HDO, MJFC, and OCM provided scientific insights, revised, and edited the manuscript; CWW, JFSF, CSA, CP, and MBDBD enrolled patients; and BKAR, ACN, and OCM supervised the project. The last 3 authors equally supervised the study and contributed with scientific insights; the order of authors was decided based on the time dedicated to the development of the work.

## Supplementary Material

Supplemental data

Supplemental data set 1

Supplemental data set 2

Supplemental data set 3

Supplemental data set 4

Supplemental data set 5

## Figures and Tables

**Table 1:**
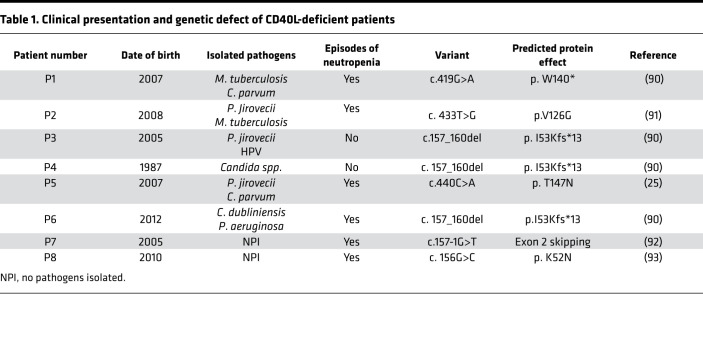
Clinical presentation and genetic defect of CD40L-deficient patients

## Version 1 07/13/2021

In-Press Preview

## Version 2 08/23/2021

Electronic publication
