# Supplementary material for: CD40L modulates transcriptional signatures of neutrophils in the bone marrow associated with development and trafficking
Source: JCI Insight. 2021 Aug 23;6(16):e148652. doi: 10.1172/jci.insight.148652 (PMC8410015; doi:10.1172/jci.insight.148652)
Supplement: Supplemental data [file jciinsight-6-148652-s119.pdf]

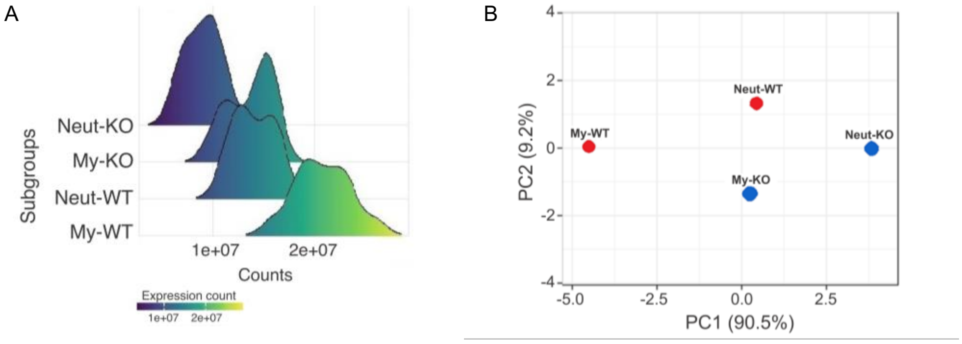

Supplementary Figure 1. Number of myeloid cells and neutrophils in the bone marrow. (**A**) Histogram and (**B**) Principal component analysis (PCA) comparing the number of BM myeloid cells and neutrophils from WT and CD40L^{-}/$^{-}$ (KO) mice.

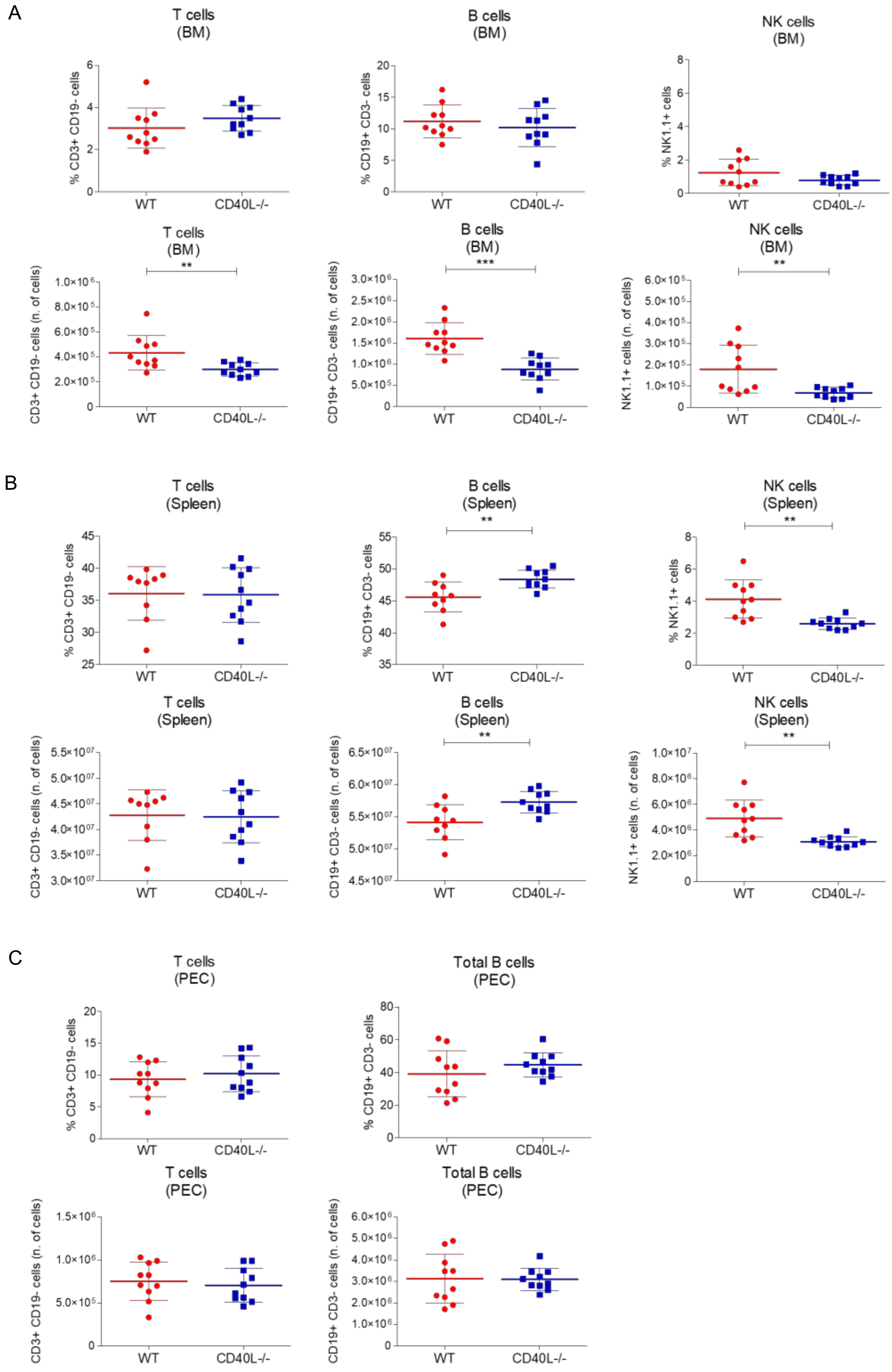

Supplementary Figure 2. Evaluation of lymphoid subpopulations in BM, spleen, and peritoneal cavity of CD40L$^{-/-}$ and WT mice. Cell percentages (top panel) of BM (**A**), spleen (**B**), and peritoneal cavity (**C**) leukocytes obtained from wild-type (WT) and CD40L$^{-/-}$ knockout (CD40L$^{-/-}$) were determined after cell staining with conjugated antibodies to identify the % of T cells (CD3^{+} C19^{-}), B cells (C19^{+} CD3^{-}), and NK cells (NK1.1^{+} CD3^{-} CD19^{-}) by flow cytometry (n=10). The number of subpopulations was obtained based on the percentage of cells compared to the cell number average cell number of each organ obtained from 19 (WT) and 13 (KO) mice. p <0.05; **p<0.01; ***p<0.0001.

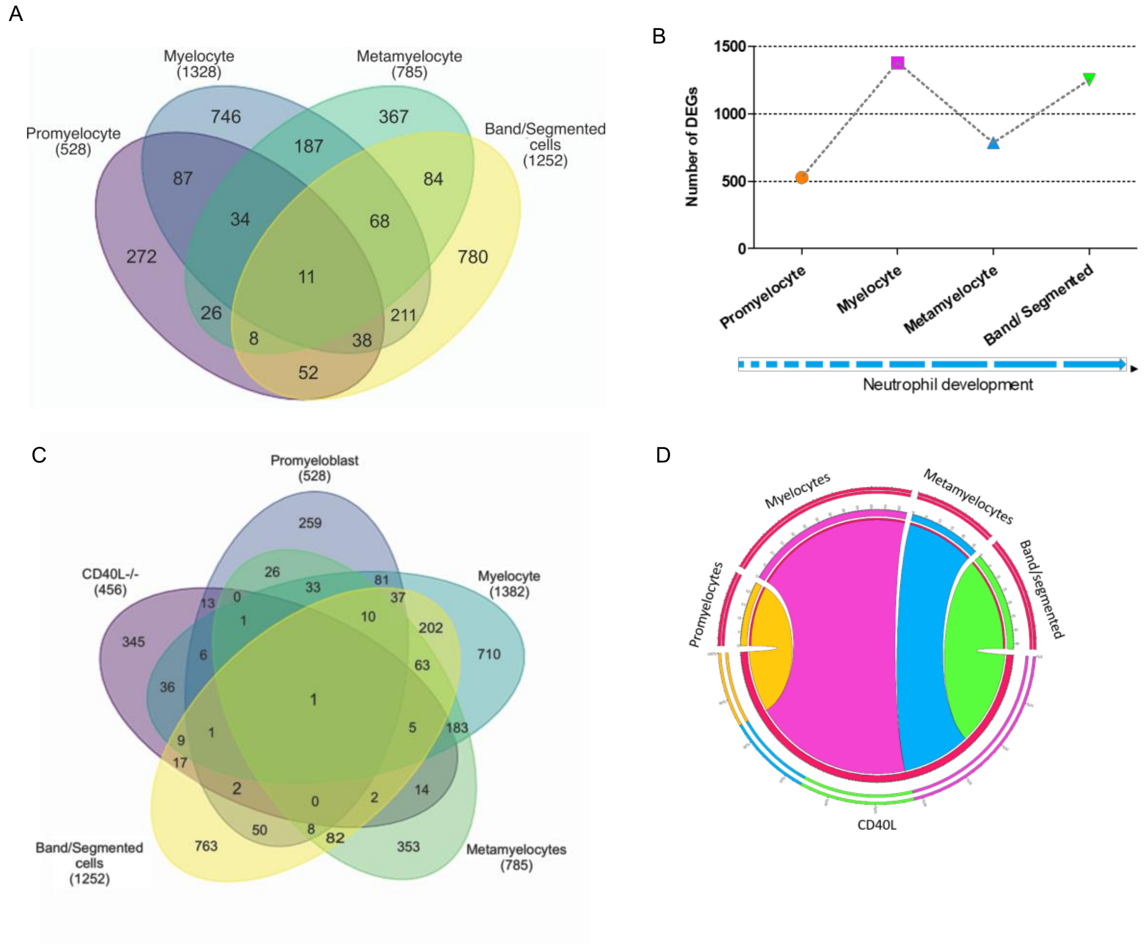

Supplementary Figure 3. Transcriptomic analysis of normal neutrophil development stages. A public available dataset extracted from a study investigating the transcriptome profile exhibited by BM neutrophils during normal developmental trajectory in WT mice was used to perform the following analysis (GSE137538). (**A**) Venn diagram showing the number of DEGs and shared genes identified comparing normal neutrophil development stages. (**B**) Gene signature of each neutrophil development stage (promyelocyte, myelocyte, metamyelocyte, and band/segmented neutrophil) was obtained by comparing the transcriptome of each developmental stage to the previous one. (**C**) Venn diagram showing normal neutrophil development stages compared to CD40L-/- DEGs. (**D**) Circle plot comparing the DEGs obtained in each developmental stage to the DEGs identified in CD40L$^{-/-}$ mice. The width of each link in the Circos plot represents the number of shared DEGs.

A

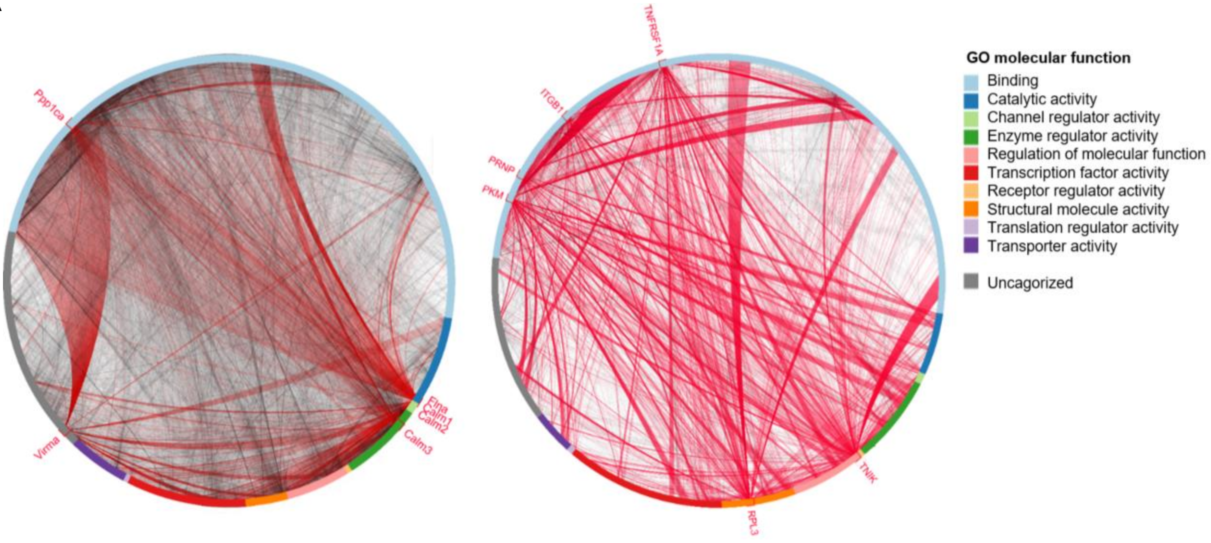

Supplementary Figure 4. Protein-protein interaction analysis. Interaction network showing the protein-protein interactions for all DEGs and Gene Ontology molecular function annotation of the genes from both CD40L$^{-/-}$ mice (left circle) and CD40L-deficient patients (right circle). Lines represent the physical protein interactions between the gene products. Nodes with red outline (and red name) represent proteins with the most connection (highest degree, and thus connecting to most of the gene products from CD40L$^{-/-}$ mice and CD40L-deficient patients) in the network. Red lines highlight their interactions. All interactions are listed in Supplemental Table 5.
